# Quadriceps autograft is a viable graft choice for arthroscopic ACL reconstruction in patients over 50 years of age

**DOI:** 10.1007/s00167-023-07367-2

**Published:** 2023-03-14

**Authors:** Amit Meena, Luca Farinelli, Christian Hoser, Elisabeth Abermann, Akshya Raj, Caroline Hepperger, Mirco Herbort, Christian Fink

**Affiliations:** 1 Gelenkpunkt - Sports and Joint Surgery, FIFA Medical Centre of Excellence, Olympiastraße 39, 6020 Innsbruck, Austria; 2 Research Unit for Orthopedic Sports Medicine and Injury Prevention (OSMI), Private University for Health Sciences, Medical Informatics and Technology, Innsbruck, Austria; 3 Fowler Kennedy Sport Medicine Clinic, Western University, London, ON Canada; 4 Clinical Orthopedics, Department of Clinical and Molecular Sciences, Università Politecnica delle Marche, Ancona, Italy; 5 Central Institute of Orthopedics, Vardhman Mahavir Medical College and Safdarjung Hospital, New Delhi, 110029 India; 6 OCM Clinic, Munich, Germany

**Keywords:** ACL, Anterior cruciate ligament, Quadriceps Graft, Over 50 years, Functional outcome

## Abstract

**Purpose:**

The purpose of this study was to evaluate the patient-reported outcomes, graft failure, quadriceps rupture and sports preference after arthroscopic ACL reconstruction in patients older than 50 years who underwent arthroscopic ACL reconstruction with a quadriceps tendon (QT) autograft.

**Methods:**

Between 2010 and 2020, prospectively collected data were obtained from an institutional database. Patients older than 50 years with primary arthroscopic ACL reconstruction and a minimum of 2 years of follow-up were included. Patients undergoing a revision ACL reconstruction or undergoing a primary ACL reconstruction using a graft other than a QT autograft, and patients with a contralateral knee injury or osteoarthritis (Ahlbäck stage 2 or higher) were excluded. A minimally invasive technique was used for QT autograft harvesting. Patients were evaluated for pre-injury and 2-year follow-up Lysholm knee score, Tegner activity level, Visual Analog Scale (VAS) for pain, graft failure, quadriceps tendon rupture, and return to sport.

**Results:**

A total of 57 patients were included in the study. The mean age of the cohort was 54.9 ± 5.2 (range 50–75). Of the 57 reconstructions, 16 (28%) were isolated ACL reconstructions, while 41 (72%) were complex reconstructions (concomitant meniscus, cartilage and/or collateral ligament injuries). At the 2-year follow-up Lysholm knee score, Tegner activity level and VAS for pain improved to pre-injury level and no significant difference was noted between pre-injury and 2-year follow-up functional scores (n.s.). No case of graft failure or quadriceps tendon rupture was reported. No significant difference was noted in the pre-injury and postoperative sports preference (n.s.) and all patients return to their desired sports activity.

**Conclusion:**

Arthroscopic ACL reconstruction by using QT autograft in highly active older patients provides satisfactory patient-reported functional outcomes and allows recovery of the pre-injury level of activity. QT autograft is a good graft option in patients older than 50 years.

**Level of evidence:**

Level IV.

## Introduction

Middle-aged patients are increasingly involved in cutting and pivoting activities and consequently increasing the incidence of anterior cruciate ligament (ACL) injuries [23]. In the past, surgical treatment was not recommended for older patients due to fear of infection, reoperation, progression towards osteoarthritis, postoperative stiffness, and residual pain [23, 25]. Therefore, conservative treatment in the form of modification of sports activities, physiotherapy and functional bracing was suggested [5, 16]. While conservative treatment is associated with satisfactory results in less active older patients, there is an increased risk of residual instability, and associated chondral and meniscal injuries in highly active patients [3, 8]. Additionally, these older active patients frequently desire to return to their pre-injury level of function and specific sport participation [16, 18, 30]. Therefore, to maintain an active lifestyle and prevent decreased knee function, ACL reconstruction is being increasingly used in active patients [27, 29]. Recent studies reported good results of ACL reconstruction in older patients and the results were comparable to young patients [2, 3, 16, 22, 27].

Despite the good results of ACL reconstruction in older patients, the ideal graft choice remains controversial. Previous studies mainly focused on ACL reconstruction with Bone-patellar tendon-bone (BPTB) and hamstring tendon (HT) autografts. However, BPTB harvesting may result in anterior knee pain, limited range of movement (ROM), and osteoarthritis (OA) of the knee [11]. On the other hand, HT autograft harvesting may cause sensory deficits due to the injury of infrapatellar branches of the saphenous nerve, compromise medial stability of the knee, and also causes weakness of internal rotation and knee flexion [12, 13]. Allograft is another graft option in elderly patients but it is associated with higher cost, risk of infectious disease transmission, delayed graft incorporation and higher failure rate [1, 7]. Therefore, in recent years, Quadriceps tendon (QT) autograft is becoming increasingly popular due to lower donor site morbidity than BPTB and HT and decreased failure rate than HT graft [4, 17, 19, 24, 31]. On the other hand, the use of QT autograft for ACL reconstruction in older patients may be responsible for quadriceps tendon rupture which is normally reported in older male patients. However, this is a very rare condition with a very low incidence of 1.37 per 100,000 cases [21].

To the best of the author’s knowledge, no study was available that used QT autograft for ACL reconstruction in older patients. Therefore, the purpose of this study was to evaluate the patient-reported outcomes, graft failure, quadriceps rupture and sports preference after arthroscopic ACL reconstruction in patients older than 50 years who underwent arthroscopic ACL reconstruction with a quadriceps tendon (QT) autograft. The hypothesis was that arthroscopic ACL reconstruction with QT autograft in older patients will provide satisfactory results without any complications in terms of graft failure and quadriceps tendon rupture. It was also hypothesized that after arthroscopic ACL reconstruction patients will be able to return to pre-injury sports preferences and activity levels.

## Materials and methods

The study was performed at Gelenkpunkt – Sports and Joint Surgery, FIFA Medical Centre of Excellence and approved by the ethics committee of the Medical University of Innsbruck (AN2015-0050). Between 2010 and 2020, prospectively collected data were obtained from an institutional database. Patients were included in the study if they fulfilled the following inclusion criteria: Primary arthroscopic ACL reconstruction using QT autograft, age 50 years and above, and had a minimum of 2-year follow-up after ACL reconstruction. The exclusion criteria were: revision ACL reconstruction, contralateral knee injuries, utilization of graft tissue other than quadriceps tendon, inflammatory arthritis or osteoarthritis (Ahlbäck stage ≥ 2), less than 2 years of follow-up and conditions that might interfere with the standard postoperative rehabilitation protocol.

Preoperatively magnetic resonance imaging (MRI) was performed to confirm ACL rupture and evaluate associated injuries. Plain radiographs (anteroposterior and lateral view) were obtained to exclude any bone injury and osteoarthritis (Ahlbäck stage ≥ 2). Plain radiographs were also obtained post-operatively to evaluate the placement of the femoral and tibial bone tunnel and to assess the correct position of the femoral fixation.

In the present study majority of the patients were operated on within 1 week of ACL injury. Therefore, the patient-reported pre-injury scores were recorded and used as a baseline rather than a pre-operative score. Patients were also evaluated at a 2-year follow-up for Lysholm knee score, Tegner activity level and VAS (visual analogue scale) for pain; graft failure, quadriceps tendon rupture and sports preference.

### Surgical technique

All ACL reconstructions were carried out by two fellowship-trained experienced surgeons. The use of QT autograft with or without bone block was not randomized rather it depended on the surgeon’s preference. One senior surgeon (CF) preferred QT autograft without bone block while the other surgeon (CH) preferred QT autograft with bone block. A minimally invasive technique was used for QT autograft harvesting as described by Fink et al. [9] and a rectangular femoral tunnel was used. Anatomic placement of the tibial and femoral tunnel was made through an anteromedial portal, the graft diameter and the tunnel diameter were equal in size. For QT autograft with bone block, the bone block was fixed in the femoral tunnel. Free EndoButton and number 2 FiberWire were used to prepare a fixed loop graft. Bioabsorbable interference screw (same size as the tibial tunnel) was used for distal fixation of graft while EndoButton (Smith & Nephew) was used for graft fixation at the femoral cortex for both types of QT autograft.

### Rehabilitation

A similar rehabilitation protocol was used in all patients. The immediate focus was to control pain and achieve full extension of the knee. After surgery patients were admitted for 2 days and during this period pain management and mobilization training were offered. Partial weight-bearing and knee flexion up to 90° was allowed with a knee brace for initial 2 weeks. After two weeks weight-bearing and range of motion were gradually increased as tolerated by the patients. Patients followed physiotherapy and rehabilitation program for 6–9 months.

## Statistical analysis

A priori power analysis was performed to determine the appropriate sample size for the study. Considering an α level with *P* = 0.05, a power of 80%, and an effect size of 0.5 it was estimated that 54 subjects would be needed for the present study.

Data were retrieved and organized using an Excel sheet (Microsoft, Redmond, WA, USA). Categorical variables were expressed in numbers and percentages (%). Continuous variables were expressed by the average and standard deviation (SD). The normal distribution of variables was verified through Shapiro–Wilk test. Variables were not normally distributed therefore; nonparametric tests were used for the comparison of variables. Specifically, the Wilcoxon’s rank test was used for paired samples. The chi-square statistic test was used to compare sports preferences before the injury and after surgery. *P* values less than 0.05 was indicative of statistically significant differences. Statistical analyses were performed with the R statistical program.

## Results

A total of 59 patients over the age of 50 years underwent primary arthroscopic ACL reconstruction using QT autograft. Two patients were lost to follow-up; thus, 57 patients were included in the study. Demographic details and characteristics of the study population can be found in Table 1. Out of 57 patients, 33 (57.9%) were females and 24 (42.1%) were males. The mean age at the time of surgery was 54.9 ± 5.2 (range 50–75). The mean time from injury to surgery was 16.6 days with the majority of patients 33 (57.9%) being operated on within 2 days of injury. In 38 (67%) patients QT autograft without bone block and in 19 (33%) patients QT autograft with a bone block was used. Of the 57 reconstructions, 16 (28%) were isolated ACL reconstructions, while 41 (72%) were complex reconstructions. Lateral and medial meniscal injuries were reported in 15 (26%) and 21 (37%) patients, respectively. Ten patients (24%) had chondral injuries that were treated by chondroplasty. Five patients reported MCL injuries, three of them were treated conservatively; 2 required repair with the suture anchor.

**Table 1:** Patient’s characteristics and associated injuries

Variables	Patient numbers = 57 Number (%)
Age, mean (SD)	54.9 (5.2)
Sex	
Female	33 (57.9%)
Male	24 (42.1%)
Days from injury to surgery, mean (SD) [range]	16.6 (12.9) [0–90]
QT autograft without bone block	38 (67%)
QT autograft with bone block	19 (33%)
Isolated ACL reconstruction	16 (28%)
Concomitant (complex) procedures	41 (72%)
Meniscal injuries	36 (63%)
Meniscectomy	
Medial	19 (33%)
Lateral	6 (11%)
Meniscal repair	
Medial	2 (4%)
Lateral	9 (16%)
Cartilage lesions	10 (18%)
MCL injuries	5 (9%)
Summer sport before ACL injury	
Cycling	16 (28%)
Running	15 (26%)
Mountain biking	7 (12%)
Others	13 (23%)
Winter sport before ACL injury	
Ski/Snowboard	52 (91%)
Others	5 (9%)
Sport after ACL injury	
Summer sport at 2-year follow-up	
Running	15 (26%)
Cycling	13 (23%)
Mountain biking	9 (16%)
Others	15 (26%)
Winter sport after ACL injury	
Ski/Snowboard	46 (81%)
Others	11 (19%)
Frequency of sports before ACL injury	
> 5 times by week	15 (26%)
2–3 times by week	36 (63%)
Irregularly	6 (11%)
Frequency of sports at 2-year follow-up	
> 5 times by week	11 (19%)
2–3 times by week	40 (70%)
Irregularly	6 (11%)

*QT* quadriceps tendon, *SD* standard deviation

Compared to pre-injury, there was no statistically significant difference (n.s.) in Lysholm knee score, Tegner activity level, and VAS for pain at the two-year follow-up (Table 2). During the follow-up period, neither graft failure nor quadriceps tendon rupture was reported in any of the patients. No case of patella fracture was reported during the QT autograft with bone block harvesting. No significant difference (n.s.) was noted in the pre-injury and postoperative sports preference and all the patients returned to pre-injury level sports activities.

**Table 2:** Patients reported outcomes measures

Variables	Pre-injury score	2-year FU score	*P* value
VAS	1.0 (1.6)	0.5 (0.9)	n.s
Tegner activity score	5.0 (2; 4–6)	5.0 (2; 4–6)	n.s
Lysholm knee score	89.9 (17.8)	93.4 (8.9)	n.s

Data are expressed as mean (standard deviation, SD) or median (interquartile range)

*FU* follow-up, *n.s* not significant

## Discussion

The most important finding of this study was that arthroscopic ACL reconstruction with QT autograft allows highly active older patients to achieve pre-injury level functional outcomes. All patients returned to desired sports activities (cutting, pivoting) with no cases of graft failure at the end of a 2-year follow-up. No case of quadriceps rupture or patella fracture was reported.

Life expectancy has increased over the years and the current median age of the European population is 44.1-year which is projected to increase by 48.2 years at the end of 2050. Therefore, a large part of the population falls in the category of 50 years and older. Sports participation has also increased in this ageing population which may result in an increased incidence of ACL injuries. ACL reconstruction has shown good results in this age group, especially in high-demand patients. ACL reconstruction is more cost-effective than conservative treatment in terms of societal and economic impacts [6, 27].

In the present study, the pre-injury Lysholm knee score was 89.9 points which increased to 93.4 points at a 2-year follow-up (n.s.). Kim et al. found improvement in Lysholm score from baseline 78.5 points to 93.0 points at 1-year follow-up in patients older than 50 years [15]. Iorio et al. used HT autograft and compared the functional outcomes between younger and older patients. They reported similar functional outcomes between the two age groups and noted that the Lysholm score improved to 94.3 points following ACL reconstruction in older patients at a 5-year follow-up [14]. Similarly, in another study, Lysholm's score improved to 93.2 points after ACL reconstruction in patients older than 60 years [29]. In the present study, Lysholm’s score improved and reached a pre-injury level which indicates that ACL reconstruction is a satisfying procedure in older active patients. The improvement in Lysholm’s score is comparable to that in previous studies.

After conservative treatment, knee instability may remain and because of this instability activity level decreases which leads to a decrease in quality of life [15]. In the current study, after ACL reconstruction the mean Tegner activity score reached to pre-injury level. Previous studies have also shown improvement in Tegner activity after ACL reconstruction [23, 30, 32]. The findings of the previous studies are comparable to the present study. After ACL reconstruction patients undergo intense rehabilitation and this may be a reason for the improvement in the activity level compared to the pre-injury level. VAS for pain also reached a pre-injury level and the VAS score improved from 1.0 to 0.5 points at a 2-year follow-up. Similar, improvement in the pain score was reported in the previous studies [8, 22].

The risk of graft failure is more common in adolescents and young adults compared to older patients [10, 26, 28]. In the present study, no case of graft failure was reported. Similar, results of graft survivorship were reported in previous studies [2, 14, 15]. Older patients are more careful after the surgery and this may be a reason for 100% graft survivorship in the current study. Along with graft failure, this study also looked for distal quadriceps tendon rupture. It is a rare condition, that usually occurs due to sudden eccentric loading of the quadriceps muscle. It is seen in middle-aged patients with a mean age of 51.1 years [21]. Therefore, many surgeons are apprehensive about using QT autograft as a graft choice in older patients. In the present study, no case quadriceps rupture was noted at the 2-year follow-up.

Sports activities are an extremely good channel for social interaction, developing relationships, and contributing to the overall sense of successful ageing [20]. The current study demonstrates that the preference for sporting activity had not changed from the pre-injury level and almost all the patients returned to desired summer sports such as running, cycling and mountain biking. While 81% of patients returned to pivoting sports such as skiing compared to 91% pre-operatively. Although there was a decrease in participation in skiing, this decrease was not significant (n.s.). In previous studies by Panisset et al. [23] and Fayard et al. [8] 83% of patients returned to sports. Toanen et al. in their study of 60-year-old patients found that 83% of patients returned to sports including regular alpine skiing [29]. While in a recent study by Ovigue et al. 86% of patients returned to any type of sport and 72.3% of patients returned to skiing [22]. They noted that one of the main reasons for a decrease in pivoting sports was fear of re-injury. The findings of these previous studies are similar to the current study.

There are a few limitations of the study. First, this was a retrospective analysis of patient-reported subjective outcome measures. Prospective comparative studies and randomized trials considering objective scores along with subjective scores should be conducted which will be of higher evidentiary value. Second, the small size of the cohort limits the generalizability of the results, therefore, the findings of the study need to be confirmed in a large sample size study. Third, small size leads to limited and underpowered statistical analysis (Type II errors). However, the present research represents one of the largest series of ACLR in patients older than 50 years old with quadriceps tendons. Fourth, lack of a control group of conservatively managed patients. However, conservative treatment is offered to low-demanding individuals who can cope with knee instability and in whom quality of life is not affected by an ACL injury. Therefore, a comparison between conservatively and surgically treated patients may lead to selection bias. Fifth, the follow-up was short to report the evolution of arthritis and to comment on sports participation in the long term, but 2 years is sufficient time to evaluate a return to sports and no case of catastrophic deterioration was noted at the 2-year follow-up. Sixth, Different surgeons may have had different indications for selecting a QT autograft and for deciding whether to harvest the graft with or without a bone block, leading to a performance bias. Seventh, baseline scores were taken during the first postoperative week which may result in recall bias; however, the majority of patients were operated on within the first week of the injury. Despite these weaknesses, the findings of the present study suggest that arthroscopic ACL reconstruction using QT autograft is a viable surgical option in appropriately selected, motivated older patients who enjoy athletic activities.

The clinical relevance of the current study is that as life expectancy has increased, so have sports participation and subsequent ACL injuries. Surgeons should be aware of all the available graft options, including the QT autograft. The findings of this study will provide insight into the treatment plan and counselling of highly active older patients.

## Conclusion

Arthroscopic ACL reconstruction by using QT autograft in highly active older patients provides satisfactory patient-reported functional outcomes and allows recovery of the pre-injury level of activity. QT autograft is a good graft option in patients older than 50 years.

## Data Availability

The datasets generated and analyzed during the current study are available from the corresponding author on reasonable request.
